# A Novel Sulfonyl-Based Small Molecule Exhibiting Anti-cancer Properties

**DOI:** 10.3389/fphar.2020.00237

**Published:** 2020-03-12

**Authors:** Abed El-Hakim El-Kadiry, Jamilah Abusarah, Yun Emma Cui, Nehme El-Hachem, Ian Hammond-Martel, Hugo Wurtele, Sini Thomas, Maryam Ahmadi, Mohammad Balood, Sébastien Talbot, Moutih Rafei

**Affiliations:** ^1^Department of Biomedical Sciences, Université de Montréal, Montreal, QC, Canada; ^2^Department of Pharmacology and Physiology, Université de Montréal, Montreal, QC, Canada; ^3^Department of Microbiology and Immunology, McGill University, Montreal, QC, Canada; ^4^Genomics Institute of Precision Medicine, American University of Beirut, Beirut, Lebanon; ^5^Department of Medicine, Université de Montréal, Montreal, QC, Canada; ^6^Maisonneuve-Rosemont Hospital Research Center, Montreal, QC, Canada; ^7^Molecular Biology Program, Université de Montréal, Montreal, QC, Canada; ^8^Department of Microbiology, Infectious Diseases and Immunology, Université de Montréal, Montreal, QC, Canada

**Keywords:** HTS, small molecule, sulfonyl compound, T cells, cancer, apoptosis

## Abstract

Phenotypic screening is an ideal strategy for the discovery of novel bioactive molecules. Using a customized high-throughput screening (HTS) assay employing primary T lymphocytes, we screened a small library of 4,398 compounds with unknown biological function/target to identify compounds eliciting immunomodulatory properties and discovered a sulfonyl-containing hit, we named InhiTinib. This compound inhibited interferon (IFN)-gamma production and proliferation of primary CD3^+^ T cells without inducing cell death. In contrast, InhiTinib triggered apoptosis in several murine and human cancer cell lines. Besides, the compound was well tolerated by immunocompetent mice, triggered tumor regression in animals with pre-established EL4 T-cell lymphomas, and prolonged the overall survival of mice harboring advanced tumors. Altogether, our data demonstrate the anti-cancer properties of InhiTinib, which can henceforth bridge to wider-scale biochemical and clinical tests following further in-depth pharmacodynamic studies.

## Graphical Abstract

Using a high-throughput screening (HTS) assay employing transgenic T cells which fluoresce upon T cell receptor (TCR) activation, we screened a small library of compounds with unknown biological function/target to identify compounds with potential immunomodulatory properties. Among the screened library, we discovered a sulfonyl-containing hit, we named. This compound inhibited interferon (IFN)-gamma production and proliferation of primary CD3^+^ T-cells without inducing cell death. In contrast, InhiTinib triggered apoptosis in several murine and human cancer cell lines. Further *in vivo* studies showed the compound is well tolerated by immunocompetent mice, triggers tumor regression in animals with pre-established EL4 T-cell lymphomas, and prolongs the overall survival of mice harboring advanced tumors.

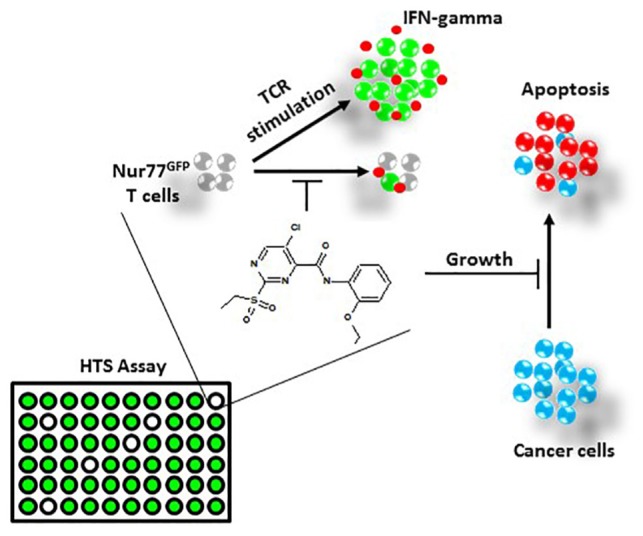

## Introduction

The discovery and development of new pharmaceutical entities is a daunting procedure employing distinct approaches. An example is target-based drug development which requires prior knowledge of a disease, its molecular targets (Macarron et al., 2011) and, arriving last, the validation of any potential therapeutic effects (e.g., inhibition of cell proliferation or induction of cell death) induced by exploited active compounds in the setting of an assay (Sittampalam et al., 2004; Malo et al., 2006). The golden standard for such assays is high-throughput screening (HTS) (Sittampalam et al., 2004), which can test the therapeutic potential of millions of structurally heterogeneous chemical entities with undefined biological properties – the goal being the assessment of phenotypic changes induced on a given target cell (Houston et al., 2008). Consistent with this claim, several FDA-approved drugs against cancer (Gefitinib, Erlotinib), HIV (Tipranavir, Maraviroc), pulmonary hypertension (Ambrisentan), diabetes (Sitagliptin), and thrombocytopenia (Eltrombopag) were initially discovered by HTS (Macarron et al., 2011) and became pharmaceutically available following subsequent biochemical optimization (Mullin, 2004).

In this study, we present a customized, highly reproducible, and read out-efficient phenotypic screening platform for the identification of bioactive compounds with onco-immunological indications. Briefly, the HTS assay uses Nur77^GFP^ mice-derived T cells whose activation drives Nur77 promoter activity and subsequent green fluorescent protein (GFP) expression. Therefore, fluorescence levels reflect T-cell receptor (TCR) activation intensity (Moran et al., 2011). As such, bioactive entities reducing fluorescence represent potential immunomodulatory hits (Fouda et al., 2017; see section “HTS Assay”). Using this HTS assay, we discovered a small compound, we named InhiTinib, exhibiting both immunomodulatory and anti-cancer properties. Although the involved molecular targets are still under investigation, InhiTinib’s dual activity might originate from its sulfonyl group, which is a known bioactive entity affecting metabolic signaling pathways and present in several FDA-approved drugs (Chen et al., 2012; Piton et al., 2018). Indeed, InhiTinib’s properties were herein elucidated in a series of *in vitro* and *in vivo* experiments which further test its safety and efficacy while highlighting its therapeutic potential.

## Materials and Methods

### Cell Lines, Mice, and Human Cord Blood

The EL4, A20, and P815 cell lines were purchased from ATCC. The BT549 and U87 cell lines were kindly provided by Dr. Annabi Borhane (Univeristé du Quebec à Montréal, QC, Canada). The B16 cell line was kindly provided by Dr. Nicoletta Eliopoulos (McGill University, QC, Canada). The MDA-MB-231 cell line was kindly provided by Dr. Koren Mann (McGill University, QC, Canada). The U-2 OS cell line was kindly provided by Dr. Hugo Wurtele (Université de Montréal, QC, Canada). Female 6–8 weeks old C57BL/6 or Balb/c mice were purchased from Jackson Laboratory. Mice were interbred and housed in a pathogen-free environment at the animal facility of the Institute for Research in Immunology and Cancer (IRIC, QC, Canada). Animal protocols were approved by the Animal Care Committee of Université de Montréal (QC, Canada). Human cord blood (CB) units were obtained from St. Justine Blood Bank (Montreal, QC, Canada) following ethics approval.

### Cell Culture and Reagents

The compound library, InhiTinib, and its related analogs were provided by the IRIC HTS platform. Because the analog ligands are proprietary, their structures are not shown here. CD3-CD28 dynabeads, CellTrace^TM^, Hoechst 33342, nerve growth factor (NGF), and MitoSox^TM^ Red were purchased from Thermo Fisher. Human and murine interferon (IFN)-gamma Quantikines were purchased from R&D System. Annexin-V, propidium iodide (PI) and anti-TUBB3 antibody were purchased from Biolegend. Lipopolysaccharide, glial-derived neurotrophic factor (GDNF), cytosine arabinoside (AraC), and the immunosuppressive agent Cyclosporin A were purchased from Sigma. Collagenase A and dispase II were purchased from Roche Applied Sciences. T-cell isolation kit and lymphoprep^®^ were purchased from StemCell Technologies. Z-VAD-FMK pan-caspase inhibitor was purchased from APExBIO. Western blot antibodies against human and murine poly(ADP-Ribose) polymerase-1 (PARP-1) (Cat. #sc-8007 and 46D11) were purchased from Santa Cruz and Cell Signaling, respectively; γH2AX (Cat. #05-636) from EMD Millipore; and H4 and tubulin (Cat. #ab6161) from Abcam SPHERO^TM^. AccuCount Particles were purchased from Spherotech, Inc.

### HTS Assay

The phenotypic screen was conducted as previously described (Fouda et al., 2017). Briefly, female Nur77^GFP^ transgenic C57BL/6 mice were purchased from the Jackson Laboratory. These mice harbor an episomal plasmid containing a cassette in which the GFP gene is under the control of the Nur77 promoter. As such, Nur77 gene expression is induced upon TCR activation. For the HTS, splenic CD3^+^ T cells were isolated using T-cell isolation kit (StemCell technologies) then stimulated with CD3–CD28 dynabeads. The beads engage TCR and CD28 co-receptor, thereby providing all required signals driving T-cell activation. Six hours later, the beads were magnetically removed and activated T cells seeded at 75 × 10^4^ cells per well in a flat-bottom 384-well plate (in a volume of 40 μl). Molecules derived from the selected library of compounds (dissolved in 0.5% v/v DMSO) were then added to each well. The plates were incubated for 24 h at 37°C and 5% CO_2_. The day of the screening, Hoechst 33342 stain solution was added to each well, and the cells were re-suspended using Biomek FX to obtain a homogenous distribution. The plates were left aside for 30 min to allow cell settling prior to fluorescence assessment using Opera Phenix high content screening system.

### Assessment of T-Cell Responses

To evaluate their responses *in vitro*, T cells were first isolated using a single cell suspension generated from the spleen of female C57BL/6 mice that was processed using the StemCell isolation kit. Isolated T cells were either activated using CD3–CD28 dynabeads or following co-culture with allogeneic Balb/c-derived dendritic cells (DCs) generated as previously described (Al-Chami et al., 2016; Tormo et al., 2017). Three days following T-cell culture, supernatants were collected to quantify IFN-gamma using commercial quantikines. For human T cells, CB units were first processed by Lymphoprep^®^ then processed using the T cell isolation kit prior to their activation using CD3–CD28 dynabeads. For the assessment of T-cell proliferation, isolated murine T cells were first labeled with CellTrace^TM^ according to the manufacturer’s instructions then co-cultured with allogeneic Balb/c-derived bone marrow-derived dendritic cells (BMDCs) in the presence or absence of InhiTinib at 3.10 μM (IC_100_). Two days later, CellTrace^TM^ dilution was assessed by flow-cytometry.

### Cell Proliferation Assay

EL4 cells were cultured overnight with increasing concentrations of InhiTinib. The following day, cell number was assessed by Flow cytometry using SPHERO^TM^ AccuCount Particles according to the manufacturer’s protocol.

### Assessment of InhiTinib-Induced Apoptosis

Cells were plated in 6-well plates at a density of 2 × 10^5^ cells/well. Adherent cells were treated with 2 ml media/well containing InhiTinib versus an equivalent volume of DMSO overnight. Cells in suspension were plated at a density of 2 × 10^5^ cells/ml in 96-well plates (U-bottom). The following day, apoptosis was evaluated using FACS CANTOII after Annexin-V/PI staining following the manufacturer’s protocol and analyzed using FlowJoV10.

### Neuronal Culture

Neurons from 8-week-old C57BL6 mice were dissociated in HEPES buffered saline completed with 1 mg/mL collagenase A and 2.4 U/mL dispase II enzymes then incubated for 70 min at 37°C. Ganglions were triturated with glass Pasteur pipettes of decreasing size in Neurobasal-A medium (2% B27 supplement), then centrifuged over a 10% BSA gradient in PBS and plated on poly-D-lysine-coated cell culture dishes. Cells were treated overnight in 1.55, 3.2, and 6.2 μM of InhiTinib in Neurobasal-A medium completed with 0.05 ng/μL NGF, 0.002 ng/μL GDNF, and 0.01 mM AraC.

### Mitochondrial Oxidative Stress Assessment in Dorsal Root Ganglion Neurons

For the evaluation of oxidative stress, cultured neurons were washed in PBS for 5 min, fixed in 4% formalin (30 min, RT), washed, and blocked for 10 min at room temperature (PBS, 0.1% v/v Triton X-100, 5% w/v BSA). In the blocking solution, neurons were labeled for 2 h at room temperature with monoclonal mouse anti-mouse TUBB3 and mitochondrial oxidative stress levels marked by MitoSox^TM^ Red. Cells were then washed three times in PBS (5 min), exposed to the secondary antibodies for 2 h in the dark, and counterstained with DAPI (1:2000). Slides were washed, coverslipped with Fluoromount G, and observed under fluorescence microscope (Nikon Eclispe Ti2). Single neuron MitoSox^TM^ Red levels were analyzed with NIS elements and reported as mean fluorescence intensity (MFI).

### Western Blotting

To further confirm the apoptosis-inducing capacity of InhiTinib, EL4 or BT549 cells were pre-treated for 24 h with 20 μM Z-VAD-FMK and then treated for another 6 h with 3.10 μM (IC_100_) of InhiTinib. Cells were then collected, washed, and lysed in 2% w/v SDS/25 mM Tris pH7.5 prior to the assessment of PAPR-1 and γH2AX by western blotting.

### Similarity Search and Target Prediction

We have mined the ChEMBL database (Gaulton et al., 2012) (>1.5 million chemical compound, ∼9,000 protein target) using an ultrafast algorithm, FPsim2, which requires RDkit program to calculate Extended-connectivity fingerprints (Landrum, 2016). A similarity threshold was set to 0.9. Compounds with a similarity index equal or greater than this threshold are considered having a similar bioactivity/target. Subsequently, bioactivities for all compounds were processed and filtered using an in-house pipeline which deploys custom R scripts. All chemical-target interactions with potency weaker than 10,000 nM were removed.

### Toxicology and Therapeutic Efficacy Studies

To evaluate the toxicity of InhiTinib, 6–8 weeks old female C57BL/6 mice (*n* = 10/group) were intraperitoneally injected with increasing doses of InhiTinib every 2 days for a total of three injections. Beside daily assessment of weight loss, peripheral blood was collected at day 6 post-initial InhiTinib administration and analyzed using Scil vet ABC Plus^+^ hematological analyzer as previously described (Al-Chami et al., 2016; Tormo et al., 2017). When animals were sacrificed, their bone marrow (BM), thymi, and spleen were isolated for flow-cytometry analysis as previously described (Al-Chami et al., 2016; Tormo et al., 2017). For efficacy studies, 6–8 weeks old female C57BL/6 mice (*n* = 10/group) were subcutaneously injected with 5 × 10^5^ syngeneic EL4 lymphoma cells. When the average tumor volume reached 100 mm^3^ (group 1) or 900 mm^3^ (group 2), animals received daily intraperitoneal injections of 100 μl Kolliphor^®^ EL solution containing 20 mg/kg of InhiTinib versus an equivalent volume of DMSO. Overall, 7 and 11 injections were given to groups 1 and 2, respectively.

### Statistical Analyses

*P*-values were calculated using ANOVA and Log-Rank statistical test where applicable. *P*-values less than 0.05 were considered significant.

## Results

### InhiTinib Impairs the Proliferation of Activated T-Cells

**FIGURE 1:**
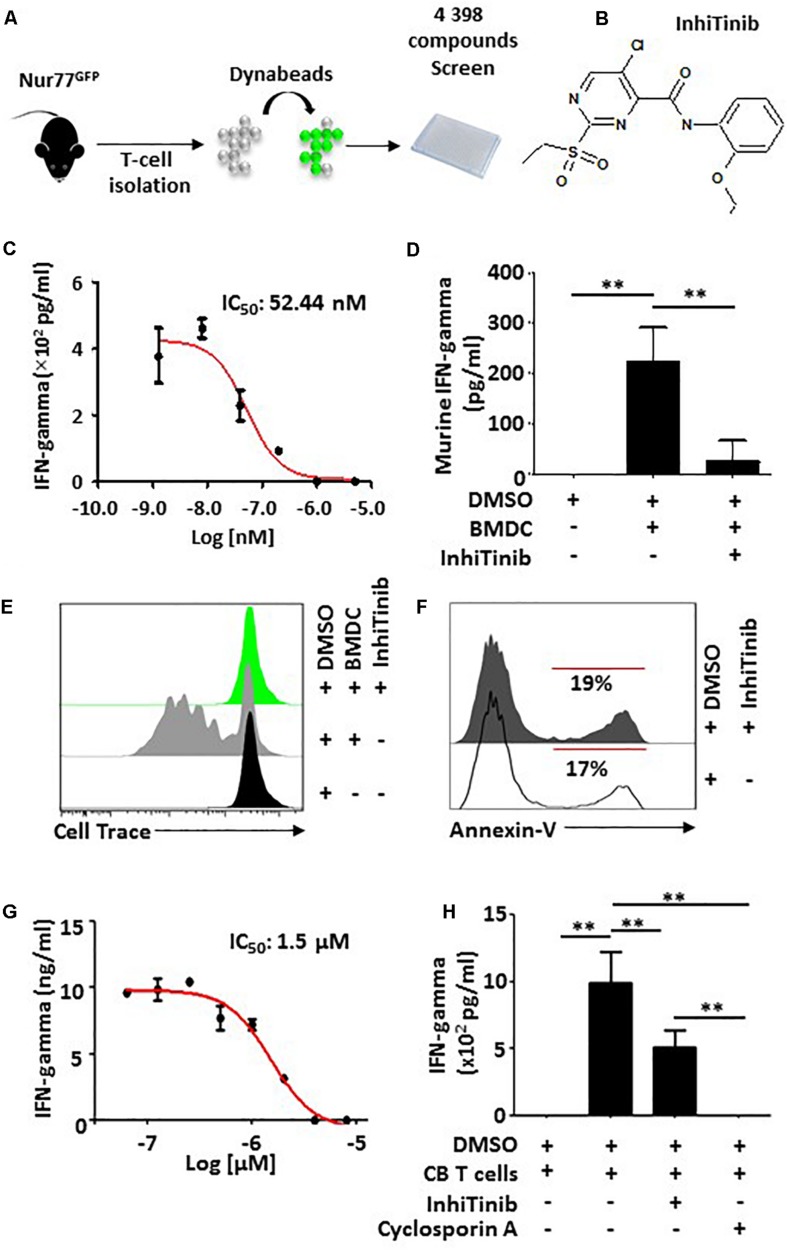
InhiTinib impairs T cell activation without inducing apoptosis. **(A)** Schematic diagram representing the experimental design undertook to identify InhiTinib. **(B)** Chemical structure of InhiTinib. **(C)** Identification of the IC_50_ dose for InhiTinib through assessment of IFN-gamma secretion by primary CD3^+^ T cells activated using CD3-CD28 dynabeads. **(D)** Quantification of IFN-gamma secretion by murine T cells cultured in a one-way MLR using allogeneic BMDCs. **(E)** Evaluating CellTrace^TM^ dilution by T cells 48 h following a one-way MLR. **(F)** Representative flow-cytometry assessment of Annexin-V on dynabeads-activated T cells treated with InhiTinib (IC_100_) vs. an equivalent volume of DMSO. **(G)** Identification of the IC_50_ dose for InhiTinib through assessment of IFN-gamma secretion by CB-derived primary human CD3^+^ T cells activated using CD3-CD28 dynabeads (*n* = 5/group). **(H)** Comparing the potency of InhiTinib to Cyclosporin A following dynabeads activation of CB-derived CD3^+^ T cells. Cyclosporin A was used at the same IC_50_ dose of InhiTinib (1.5 μM). For panels **C,H**, *n* = 5/group with ^∗∗^*P* < 0.01.

To create an efficient strategy for the discovery of active compounds with immunoregulatory properties, we designed a phenotypic HTS assay employing transgenic Nur77^GFP^ mice-derived T lymphocytes which fluoresce upon TCR activation (Figure 1A; Fouda et al., 2017). Using a preselected mini-library of 4 398 compounds representing over 150,000 commercially available small molecules, we uncovered novel (and previously functionally uncharacterized) compounds with T cell-modulating properties reflected by fluorescence signal dimming (Fouda et al., 2017). One specific hit [5-chloro-*N*-(2-ethoxyphenyl)-2-(ethylsulfonyl)-4-pyrimidinecarboxamide], we named InhiTinib (Figure 1B), exhibited powerful inhibitory effects on IFN-gamma production by activated murine T cells with an IC_50_ of 52.44 nM (Figure 1C). We also found that InhiTinib blocks IFN-gamma production from T cells co-cultured with allogeneic BMDCs in a one-way mixed lymphocyte reaction (MLR) (Figure 1D) and inhibits T-cell proliferation in a CellTrace dilution assay (Figure 1E) without inducing apoptosis (Figure 1F). Albeit less potent than Cyclosporin A, InhiTinib decreases IFN-gamma production from activated CB-derived human CD3^+^ T cells (IC_50_ = 1.5 μM; Figures 1G,H).

### InhiTinib Triggers Apoptosis in Various Cancer Cells

**FIGURE 2:**
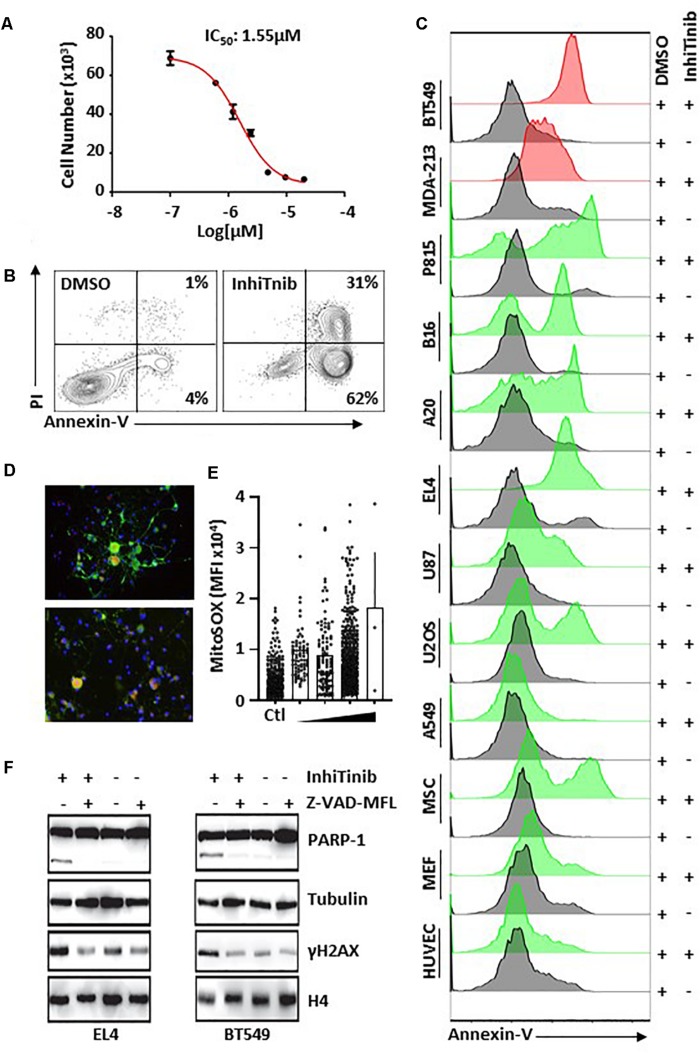
Characterizing the pro-apoptotic properties of InhiTinib on cancer cells. **(A)** Identification of the IC_50_ dose for InhiTinib using a proliferation assay conducted on the EL4 lymphoma cell line (*n* = 5/group). **(B)** Representative flow-cytometry analysis of Annexin-V/PI positive EL4 cells in response to InhiTinib treatment at 3.10 μM (IC_100_) or equivalent volume of DMSO. **(C)** Representative flow-cytometry assessment of Annexin-V on various cancer and primary cells treated with the EL4-specific IC_100_ dose identified for InhiTinib. Cell lines represented by red histograms have a mutated *p53*. **(D)** Pictograms depicting ROS levels (in red) using TUBB3^+^ dorsal root ganglion neuros (in green) following an overnight treatment with InhiTinib at 3.1 μM. **(E)** MFI quantification of ROS by microscopy. **(F)** Representative western-blot evaluating the activation of PARP-1 and γH2AX induction. Z-VAD-MFL was used to inhibit caspase activation. For illustrative purposes, blot images were cropped. A full scan of the original gels is found among Supplementary Material.

Knowing that several anti-inflammatory drugs such as NSAIDs and corticosteroids elicit anti-cancer effects (Rayburn et al., 2009), and bearing in mind the anti-cancer properties of sulfonyl groups (Casini et al., 2002), we then sought to assess whether InhiTinib displays anti-cancer activity. For this purpose, we treated the EL4 lymphoblastic cell line, a well-established model of T-cell lymphoma (Al-Ejeh et al., 2007), with InhiTinib and observed impaired cell proliferation with an IC_50_ of 1.55 μM (Figure 2A). Interestingly, the noticeable growth inhibition was due to apoptosis as almost all (93%) of InhiTinib-treated EL4 cells stained positive for Annexin-V/PI (Figure 2B). In addition, flow-cytometry analyses of apoptosis by Annexin-V staining shows that an overnight InhiTinib treatment at a dose of 3.10 μM (the IC_100_ dose for EL4), in contrast to DMSO, promotes death of murine A20 B-cell lymphoma, B16 melanoma, P815 mastocytoma, human breast cancer cell lines BT549 and MDA-MB-231, U87 glioblastoma, and U-2 OS osteosarcoma (Figure 2C). The A549 lung carcinoma was the only cell line resistant to InhiTinib (Figure 2C). When tested on normal cells, however, InhiTinib triggered the death of primary mesenchymal stromal cells (MSCs), whereas murine embryonic fibroblasts (MEFs) and human umbilical vascular endothelial cells (HUVECs) were resistant (Figure 2C). Noteworthy, treatment of primary dorsal root ganglion neurons with increasing doses of InhiTinib led to an increase in reactive oxygen species (ROS) production as shown by MitoSox^TM^ Red staining (Figures 2D,E). To further confirm that the compound triggers apoptosis, we assessed the levels of processed PARP-1, a marker of apoptosis-associated caspase activation (Javle and Curtin, 2011), and induction of γH2A histone family member X (γH2AX), a well-known marker of DNA damage (Sedelnikova et al., 2002). Treatment of EL4 (murine) or BT549 (human) tumor cells with InhiTinib caused the processing of PARP-1 and induction of γH2AX. Co-treatment with Z-VAD-MFL, an irreversible pan-caspase inhibitor, reversed these effects (Figure 2F).

### InhiTinib Is Well Tolerated *in vivo* and Displays Potent Anti-neoplastic Effects

**FIGURE 3:**
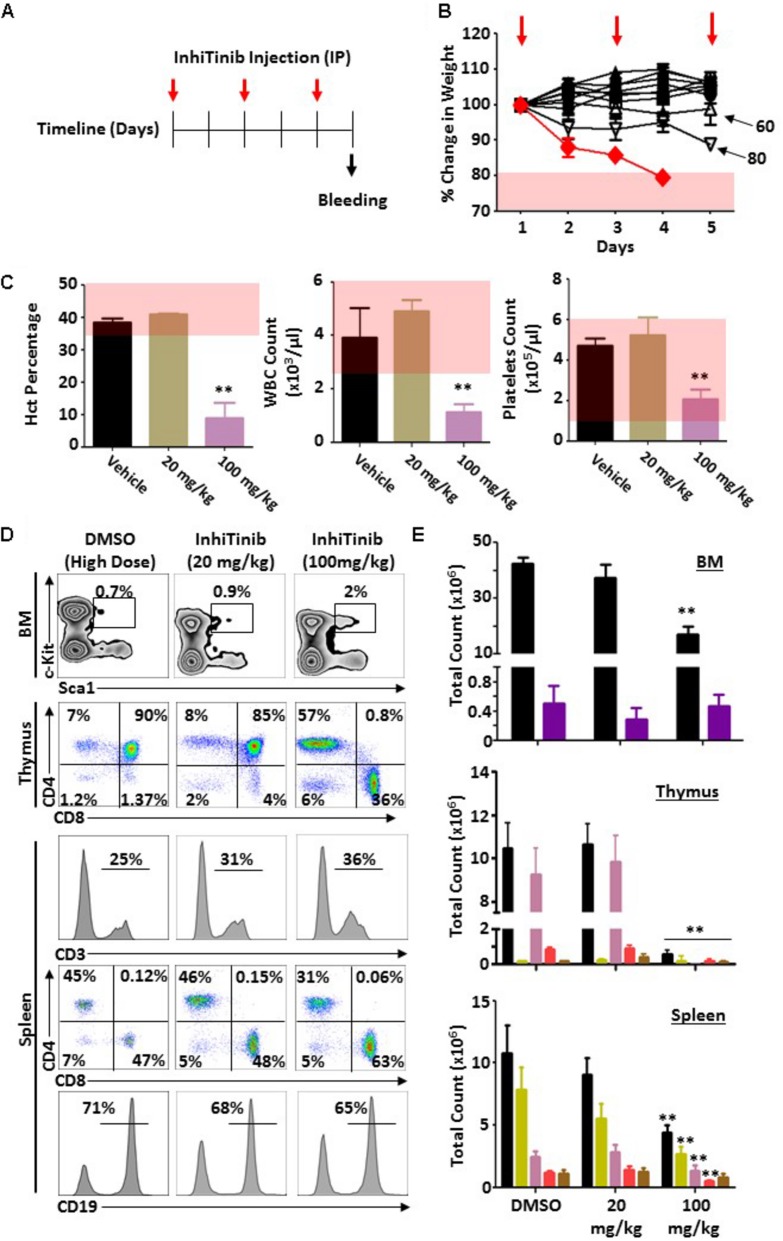
Evaluating the safety profile of InhiTinib in immunocompetent mice. **(A)** Schematic diagram representing the timeline used for the toxicity study. Red arrows depict InhiTinib administration. **(B)** Daily assessment of weight loss in animals receiving various doses of InhiTinib. The red line represents the 100 mg/kg dose whereas the pink shaded area reflects a weight loss superior to 20% of the initial animal weight. InhiTinib is represented by the red arrows. **(C)** Assessment of three CBC parameters in animals treated with the vehicle, DMSO, or InhiTinib at 20 versus 100 mg/kg. The pink shaded area represents the physiological levels for each parameter. **(D)** Representative flow-cytometry analysis of the BM, thymic, and splenic compartments in response to DMSO and low (20 mg/kg) or high (100 mg/kg) InhiTinib doses. **(E)** Quantification of the various hematopoietic cells assessed in panel D. For the BM compartment, all hematopoietic cells are shown in black whereas the hematopoietic stem cells (Lin^–^ Sca1^+^c-kit^+^) are in purple. For thymic analysis, overall thymocytes are shown in black, double-negative thymocytes in yellow, double-positive thymocytes in pink, CD4^+^ thymocytes in red, and CD8^+^ thymocytes in brown. For the spleen analysis, overall splenocytes are depicted in black, CD19^+^ B cells in yellow, CD3^+^ T cells in pink, CD8^+^ T cells in red and CD4^+^ T cells in brown. For panels D and E, *n* = 6/group with ^∗∗^*P* < 0.01.

**FIGURE 4:**
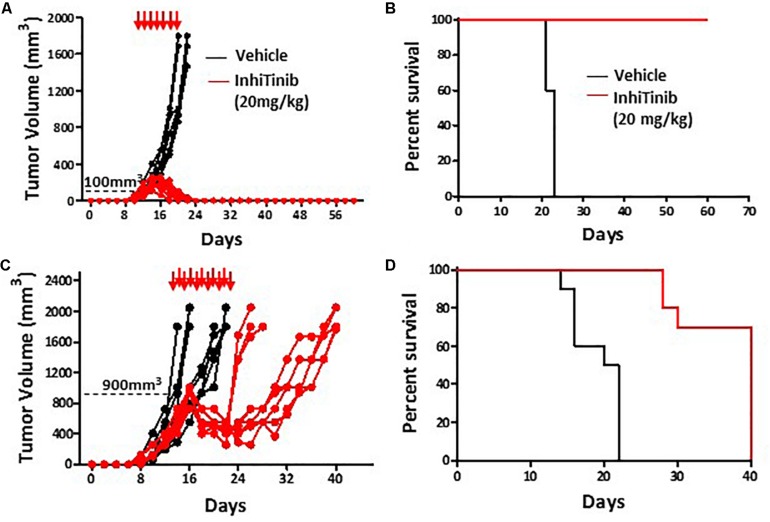
InhiTinib displays potent anti-neoplastic effects. Tumor volume **(A)** and survival assessment **(B)** of animals with small pre-established EL4 lymphoma tumors undergoing vehicle versus InhiTinib dosing at 20 mg/kg. **(C,D)** Same as A and B but using animals with larger tumor masses. For panels A-D, *n* = 10/group.

Prior to assessing the therapeutic potency of InhiTinib, we first investigated its safety profile *in vivo*. Henceforth, the sulfonyl-based compound was administered to mice thrice, specifically at days 1, 3, and 5 using doses ranging from 2 to 100 mg/kg (Figure 3A). Meanwhile, changes in body weight were recorded daily. As shown in Figure 3B, mice tolerated InhiTinib up to a dose of 80 mg/kg with a constant 15% margin of weight variation. A major drop in weight along with the appearance of moribund signs occurred at doses greater than 80 mg/kg (Figure 3B). At day 6, mice were bled, and features of complete blood count (CBC) were measured to account for hematological safety validation. Compared to the vehicle-treated group, a low InhiTinib dose (20 mg/kg) did not perturb the levels of hematocrit, white blood cells (WBC), and platelet counts, whereas 100 mg/kg of InhiTinib induced massive reduction in these parameters (Figure 3C). Besides, analyses of primary and secondary lymphoid tissues revealed a significant reduction in hematopoietic and lymphatic cell counts using a high dose of InhiTinib (100 mg/kg), whereas a lower dose (20 mg/kg) maintained normal counts of BM hematopoietic cells (c-kit^+^ Sca1^+^), thymocytes, and splenocytes (Figures 3D,E). Based on the preceding, we used the dose of 20 mg/kg for subsequent efficacy studies. Therein, the anti-cancer activity of InhiTinib *in vivo* was assessed using two groups of naïve immunocompetent C57BL/6 mice whose different-size tumors were tracked in response to daily intraperitoneal injections of InhiTinib (20 mg/kg). Syngeneic tumors were pre-established in immunocompetent mice until they reached an average size of 100 mm^3^ (group 1) and 900 mm^3^ (group 2) prior to treatment (Figures 4A,C). The dosing regimen for group 1 consisted of 7 injections in total, while the second group received 11 injections. In sharp contrast to the vehicle-treated group, InhiTinib administration to group 1 induced complete regression of pre-established tumors (Figure 4A) with no relapses throughout the time frame of the study (Figure 4B). Conversely, tumor volume in group 2 was reduced by an average of 50% during InhiTinib administration but relapsed at the end of the treatment (Figure 4C) resulting in the death of all treated animals by day 40 (Figure 4D).

## Discussion

Our work validates the feasibility of a Nur77^GFP^-based HTS platform for the identification of chemical entities with immunomodulatory properties (Fouda et al., 2017). Whereas InhiTinib impairs murine and human T-cell activation and proliferation, it triggers apoptosis in cancer cells suggesting different and/or cell-specific targets at play (Graphical Abstract). Indeed, most of the studied murine and human cancer cell lines underwent apoptosis upon InhiTinib treatment, which is clinically indicative of the wide, yet finite, anti-tumorigenic potential of this molecule. Furthermore, we showed that the DNA lesions induced by InhiTinib were mostly due to caspase activation rather than genotoxicity, thus reinforcing the safety profile of the compound (Rogakou et al., 2000; Nowsheen and Yang, 2012). However, whether caspase activation ensues in response to ROS induction (Redza-Dutordoir and Averill-Bates, 2016), endoplasmic reticulum stress (Sano and Reed, 2013), signaling modulation (Wilson et al., 2009), re-activation of mutated p53 (Bauer et al., 2016), mitochondrial outer membrane permeabilization (Kalkavan and Green, 2018), caspase-independent pathways (Kroemer and Martin, 2005), or other modes of action as predicted using a structure similarity search (Supplementary Table 1) needs further validation. Likewise, complete pharmacodynamic characterization of the molecule, especially its putative target(s) in immune and cancer cells, is ongoing, and few targets predicted through the similarity search (Supplementary Table 1) are under testing. In the same context, we believe that the sulfonyl group might impart the bioactivity of InhiTinib. In fact, sulfonyls are present in several established drugs known to antagonize multiple enzymes and receptors partaking in cellular processes such as metabolic regulation, angiogenesis, and tumor progression (Casini et al., 2002; Chen et al., 2012). Accordingly, InhiTinib may target: (i) the unfolded protein response by binding to heat shock factor 1 protein, (ii) the epigenome of cancer cells via histone methyltransferases modulation, and/or (iii) reduce antioxidant defenses by inhibiting thioredoxin reductase activity. Further studies are, therefore, warranted to decipher InhiTinib’s exact mode of action.

When tested *in vivo*, we found that InhiTinib is well tolerated up to 80 mg/kg. Ultimately, a dose of 20 mg/kg was chosen as it induced no weight fluctuations while maintaining the physiological levels of hematocrit, WBC, and platelets. As such, InhiTinib administration to immunocompetent mice with small pre-established autologous lymphomas totally prevented tumor growth and cured all animals. Since InhiTinib, as aforementioned, strongly inhibits T cells, any effect mediated by tumor-infiltrating lymphocytes on autologous tumor regression is excluded. Conversely, the response of animals with larger tumors (900 mm^3^) was less efficient indicating that the use of InhiTinib at a safe dose of 20 mg/kg is therapeutically efficient, yet its efficiency correlates negatively with tumor size at the time of treatment initiation.

Overall, InhiTinib depicts promising *in vitro* and *in vivo* anti-cancer properties, promoting caspase-dependent apoptosis in various cancer cell lines and reducing lymphoma tumor size in animals. How apoptosis ensues in response to InhiTinib and its putative target(s) in cancer/immune cells thereof are still under assessment. Plus, further biochemical and formulation studies are expected to improve its specificity and effectiveness against tumor cells. In fact, we recently identified two InhiTinib-related compounds (57695 and 57902) of the same chemical class (alkyl sulfonyl pyrimidines) with pronounced pro-apoptotic effects (Supplementary Figure 1A). Although these analogs differ slightly in their chemical structure, they all contain the sulfonyl functional group. Interestingly, compound 57695 triggered EL4 death *in vitro* without inhibiting IFN-gamma production from activated primary T cells (Supplementary Figure 1B). Thus, generating additional analogs is expected to lead to the identification of molecules with improved potency and specificity. Notable, although small molecules are therapeutically pertinent due to (i) higher tumor penetration in comparison with large molecules, (ii) amenability of formulation for oral administration, and (iii) low immune reactivity, they exert rapid short-lived responses (Wang et al., 2019). Arguably, this might not be an obstacle, as small bioactive molecules can be combined with immune-checkpoint inhibitors such as anti-PD1 or anti-CTLA4, especially against larger pre-established tumors (Zhan et al., 2016). Altogether, InhiTinib stands-out as a candidate for large scale research and development to fine-tune its anti-cancer effects and potentially harbor them in clinical research.

## Availability of Materials

The authors will make available the protocols, analytic methods, and study material upon request.

## Data Availability Statement

The raw data supporting the conclusions of this article will be made available by the authors, without undue reservation, to any qualified researcher.

## Ethics Statement

The studies involving human participants were reviewed and approved by the Comité d’éthique de la recherche (CER) CHU Sainte-Justine, Montreal, QC, Canada. The patients/participants provided their written informed consent to participate in this study. The animal study was reviewed and approved by the Animal Care Committee of Université de Montréal.

## Author Contributions

AE-K, JA, and YC designed and performed most *in vitro* and *in vivo* experiments. NE-H conducted the bioinformatics analysis. IH-M and HW designed the western blot experiments. STh, MA, and STa conducted the neuron-based studies. MB ran an apoptosis assay. MR conceived and supervised the project, analyzed data and wrote the manuscript.

## Conflict of Interest

The authors declare that the research was conducted in the absence of any commercial or financial relationships that could be construed as a potential conflict of interest.

## Abbreviations

γH2AX: γ H2A histone family member X
AraC: cytosine arabinoside
BM: bone marrow
BMDCs: bone marrow-derived dendritic cells
CB: cord blood
CBC: complete blood count
DCs: dendritic cells
GDNF: glial-derived neurotrophic factor
HTS: high-throughput screening
HUVECs: human umbilical vascular endothelial cells
IFN: interferon
MEFs: murine embryonic fibroblasts
MFI: mean fluorescence intensity
MLR: mixed lymphocyte reaction
MSCs: mesenchymal stromal cells
NGF: nerve growth factor
PARP-1: poly(ADP-Ribose) polymerase-1
PI: propidium iodide
ROS: reactive oxygen species
TCR: T-cell receptor
WBC: white blood cells.
