# Supplementary material for: A Novel Sulfonyl-Based Small Molecule Exhibiting Anti-cancer Properties
Source: Front Pharmacol. 2020 Mar 12;11:237. doi: 10.3389/fphar.2020.00237 (PMC7081885; doi:10.3389/fphar.2020.00237)
Supplement: Supplementary file 1 [file Image_1.pdf]

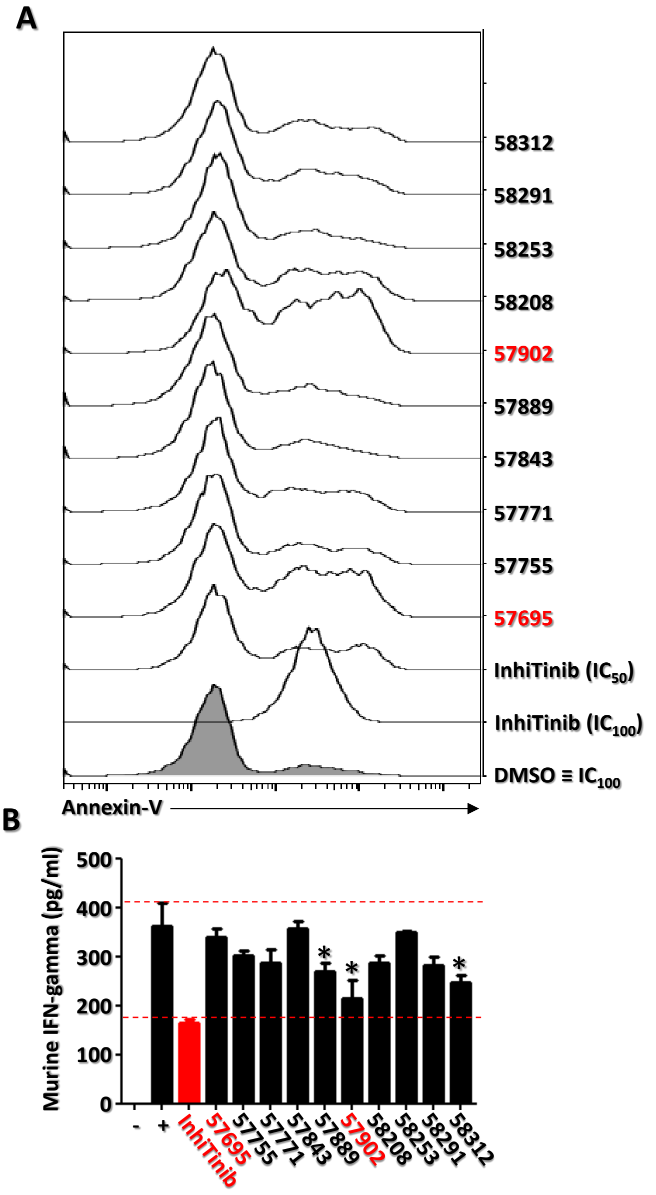

Supplementary figure 1. Assessment of InhiTinib analogs on EL4 apoptosis and T-cell activation. **A)** Representative flow-cytometry analysis of EL4 apoptosis following treatment using the IC_{50} dose identified for InhiTinib. **B)** Evaluating IFN-gamma production from activated $CD3^+$ T cells treated with the same analogs and doses shown in panel A. The red dotted lines represent the compounds range of action. For panels A and B, n=6/group with *P<0.05.
