# Supplementary material for: A Novel Sulfonyl-Based Small Molecule Exhibiting Anti-cancer Properties
Source: Front Pharmacol. 2020 Mar 12;11:237. doi: 10.3389/fphar.2020.00237 (PMC7081885; doi:10.3389/fphar.2020.00237)
Supplement: Supplementary file 2 [file Table_1.DOCX]

**Supplementary Table 1. InhiTinib similarity search and target prediction**

| **CHEMBL ID** | **MW (g/mol)** | **Standard Value** | **Target Name** | **Target Organism** |
| --- | --- | --- | --- | --- |
| CHEMBL1463077 | 355.8 | 1412.5 | Nuclear receptor ROR-gamma | *Mus musculus* |
| CHEMBL1463077 | 355.8 | 8042 | Heat shock factor protein 1 |  |
| CHEMBL1446073 | 325.78 | 1849.3 | Serine/threonine-protein kinase mTOR |  |
| CHEMBL1446073 | 325.78 | 19952.6 | Cellular tumor antigen p53 |  |
| CHEMBL1418643 | 341.78 | 35481.3 | Histone-lysine N-methyltransferase, H3 lysine-9 specific 3 |  |
| CHEMBL1412396 | 417.87 | 6966 | Microphthalmia-associated transcription factor | *Homo sapiens* |
| CHEMBL1404578 | 447.9 | 5868 | Type-1 angiotensin II receptor |  |
| CHEMBL1446073 | 325.78 | 7943.3 | Thioredoxin glutathione reductase |  |
| CHEMBL1557832 | 465.89 | 12589.3 | Microtubule-associated protein tau |  |
| CHEMBL1307050 | 415.9 | 13700 | Kappa opioid receptor |  |
| CHEMBL1353013 | 431.9 | 1976 | Streptokinase A | *Streptococcus pyogenes* serotype M1 |
