# Supplementary figures and images for: A Novel Sulfonyl-Based Small Molecule Exhibiting Anti-cancer Properties
Source: Front Pharmacol. 2020 Mar 12;11:237. doi: 10.3389/fphar.2020.00237 (PMC7081885; doi:10.3389/fphar.2020.00237)

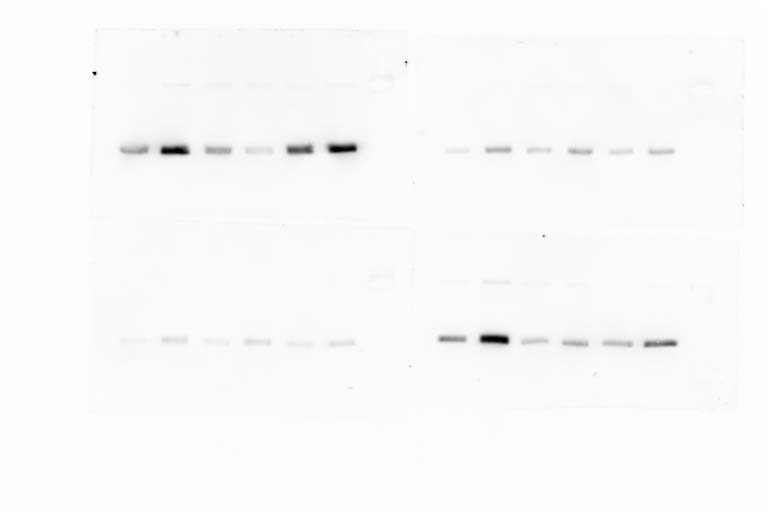

Supplement: Supplementary file 3 [file Data_Sheet_1.ZIP › yH2AX_BT549_bottom right.jpg]

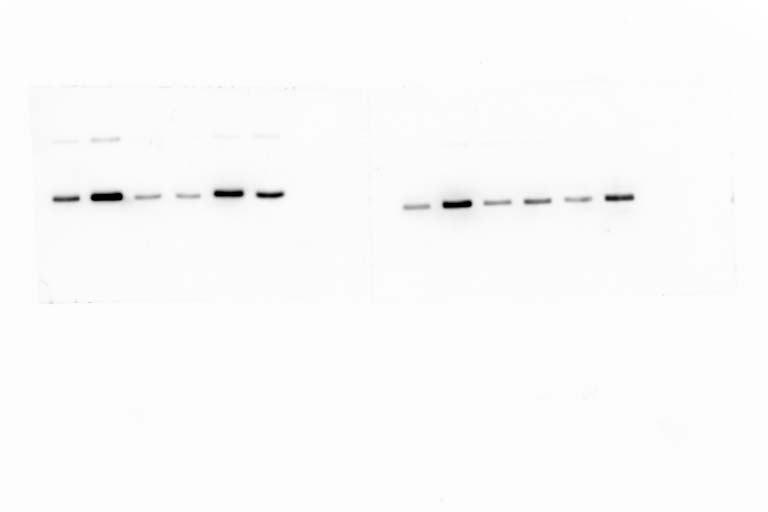

Supplement: Supplementary file 3 [file Data_Sheet_1.ZIP › yH2AX_EL4_right.jpg]

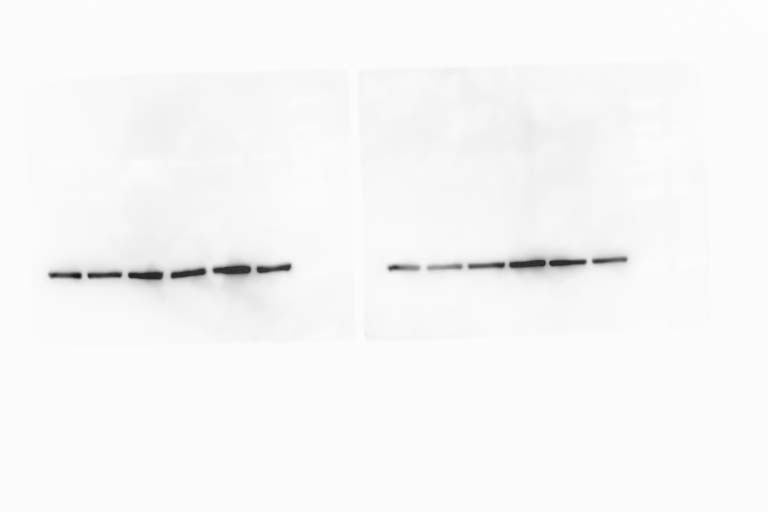

Supplement: Supplementary file 3 [file Data_Sheet_1.ZIP › Tubulin_EL4 right_BT549 left.jpg]

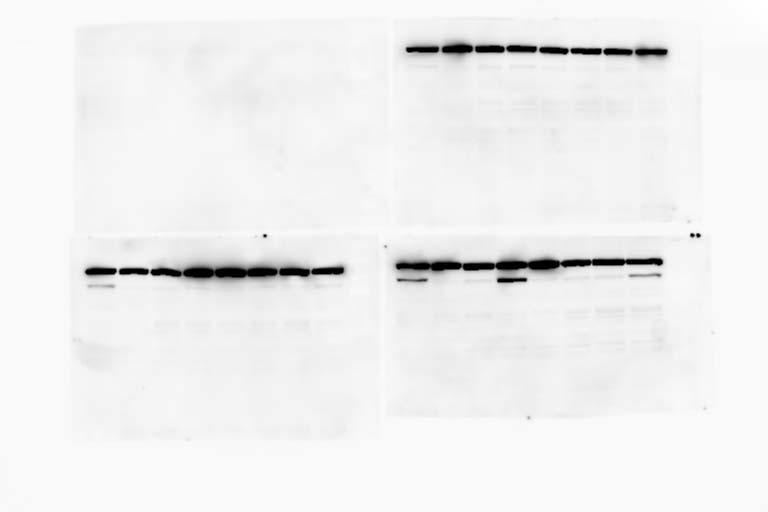

Supplement: Supplementary file 3 [file Data_Sheet_1.ZIP › PARP_BT549_bottom right.jpg]

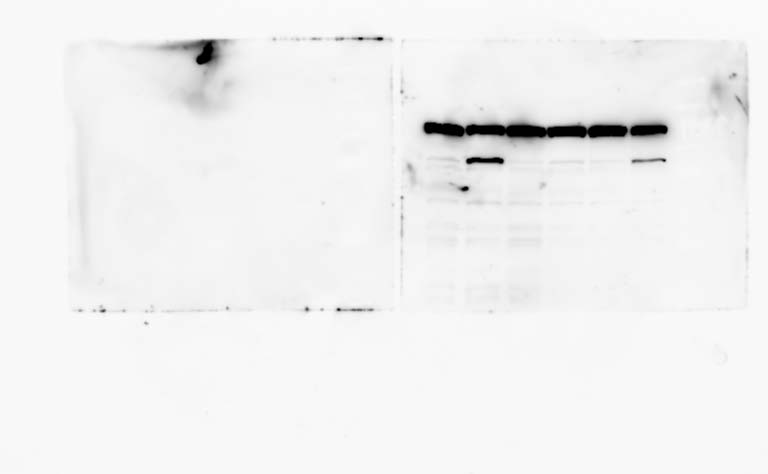

Supplement: Supplementary file 3 [file Data_Sheet_1.ZIP › PARP_EL4_right.jpg]

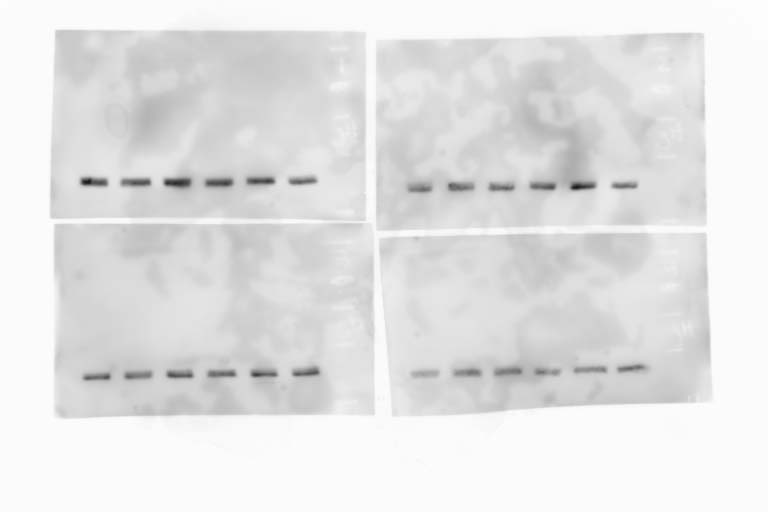

Supplement: Supplementary file 3 [file Data_Sheet_1.ZIP › H4_BT549_top left.jpg]

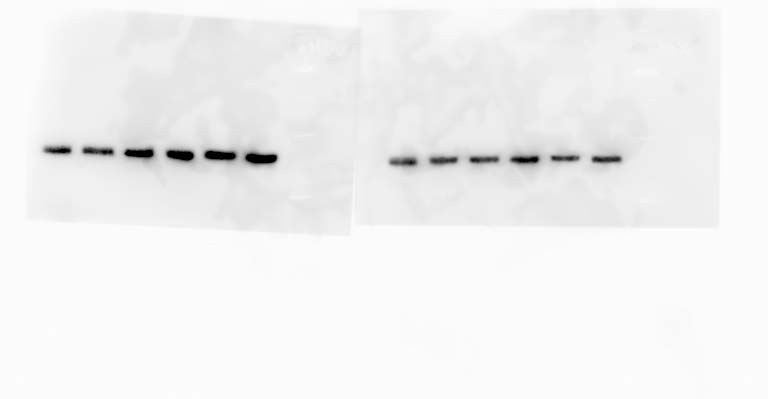

Supplement: Supplementary file 3 [file Data_Sheet_1.ZIP › H4_EL4 right.jpg]
